# Relation between FCGRIIB rs1050501 and HSV-1 specific IgG antibodies in Alzheimer’s disease

**DOI:** 10.1186/s12967-020-02495-6

**Published:** 2020-08-28

**Authors:** Andrea Saul Costa, Simone Agostini, Franca Rosa Guerini, Roberta Mancuso, Mario Clerici, Janardan P. Pandey

**Affiliations:** 1 IRCCS Fondazione Don Carlo Gnocchi, Milan, Italy; 2 Department of Pathophysiology and Transplantation, University of Milano, Milan, Italy; 3 Department of Microbiology and Immunology, Medical University of South Carolina, Charleston, SC USA

**Keywords:** Herpes Simplex Virus Type 1 (HSV-1), Alzheimer’s Disease, Mild Cognitive Impairment, Rehabilitation, IgGs, FCGRIIB

## Abstract

**Background:**

Alzheimer’s Disease (AD) is a chronic neurodegenerative disorder characterized by extracellular plaques, intracellular neurofibrillary tangles and neuronal loss in the central nervous system (CNS). Pathogens are suspected to have a role in the development of AD; herpes simplex virus type 1 (HSV-1), in particular, is suggested to be a risk factor for the disease. The gamma receptor for the Fc portion of IgG molecules (FCGRs) plays a crucial role in regulating immune responses, and among FCGRs, FCGRIIB is endowed with an inhibitory function. Notably, the rs1050501 polymorphism of *FCGRIIB* gene associates with autoimmune diseases and with neuronal uptake and interneuronal accumulation of amyloid beta in animal AD models.

**Methods:**

Genotype and allelic distribution of *ApoE4* and *FCGRIIB* rs1050501 were evaluated in a case–control population of 225 AD patients, 93 MCI individuals and 201 sex and age matched healthy controls (HC). HSV-1 total IgG titers and IgG subclasses were detected and quantified in a subgroup of the main study population by ELISA.

**Results:**

Genotype and allelic distribution of *FCGRIIB* was comparable in the study population. HSV-1-specific antibody titers were significantly higher in AD and MCI compared to HC (p < 0.01 for both); IgG3 titers, in particular, were increased in MCI compared to AD (p = 0.04). Analyses of possible correlations between the *FCGRIIB* rs1050501 genotype polymorphism and IgG subclasses showed that the presence of IgG3 was more frequent in MCI carrying the *FCGRIIB* TT (94.1%) compared to those carrying the CT genotype (63.6%) (p = 0.03).

**Conclusion:**

Results herein show an association between humoral immune response against HSV-1 and *FCGRIIB* rs1050501 genetic variation in the first stage of the disease.

## Background

Alzheimer’s Disease (AD) is an inflammatory chronic neurodegenerative disease characterized by a progressive deterioration in global intellectual ability that interferes with social and occupational performance [1]. Mild cognitive impairment (MCI), on the other hand, is an intermediate condition between physiological neurocognitive aging and AD [1, 2]. Over time, MCI can convert to AD with an estimated conversion rate of 10-to-15% [3]. The etiopathogenesis of AD likely includes both genetic and environmental factors. Among genetic factors *Apolipoprotein E* (APOE) is the only confirmed risk factor for the disease, but other genes [4], including those of the *SNARE* complex [5, 6], *PILRA* [7] and *TREM2* [8] are strongly suggested to play a role in AD. Pathogens are also suspected to be involved in AD [9]; herpes simplex virus type 1 (HSV-1), in particular, is a likely culprit [10–12].

HSV-1-specific immune response is classically mediated by antibodies (Abs), thus, the gamma receptor for the Fc portion of IgG Abs (FCGRs) forms immune complexes and activates the effector arm of the immune system [13]. Six different FCGRs are known in humans: FCGRI, FCGRIIA, FCGRIIB, FCGRIIC, FCGRIIIA, and FCGRIIIB [14]. FCGRIIB is the only FCGR endowed with inhibitory functions [15], and FCGRIIB impairments are associated with inflammatory conditions [14]. The human *FCGRIIB* gene, located on 1q23.3, includes a number of single nucleotide polymorphisms (SNPs); amongst the nonsynonymous SNPs, the T-to-C transition in exon 5 (rs1050501), which leads to a replacement of isoleucine at position 232 by threonine (FCGRIIB-I232T variant), is relatively frequent and correlates with autoimmune disease [16–19]. HSV-1 evasion from the immune response can be mediated by the expression of a viral receptor, homologous to human FCGR, which binds all human IgG subclasses, with the exception of IgG3 [20]. Susceptibility to HSV-1 infection was shown to associate with *FCGRIIIA* polymorphisms [21], but the possible role of *FCGRIIB* variants in HSV-1 infection has not been explored. Importantly, results obtained in the animal model of AD showed that the rs1050501 polymorphism of *FCGRIIB* results in neuronal uptake and interneuronal accumulation of amyloid beta [22].

We evaluated possible correlations between the HSV-1-specific humoral immune response and *FCGRIIB* rs1050501 SNP in a cohort of Italian AD, MCI, and sex- and age-matched Healthy Control (HC) subjects.

## Methods

### Patients and controls

Five-hundred-nineteen individuals were included in the study: 225 Alzheimer’s Disease (AD) patients, 93 Mild Cognitive Impairment (MCI) individuals, and 201 sex and age matched Healthy Controls (HC). All subjects were recruited by the Rehabilitative Neurology Unit of the IRCCS Santa Maria Nascente, Don Gnocchi Foundation, in Milan, Italy. Patients were diagnosed as probable AD according to the NINCDS-ADRDA criteria [1], or as MCI according to Petersen and Grundman criteria [23, 24]. Patients were excluded if they suffered from malnutrition or vitamin deficiency syndromes, and recent introduction or dose modification of the following pharmacological treatments: cholinesterase inhibitor, nemantine, antidepressant or antipsychotic drugs.

The study conformed to the ethical principles of the Declaration of Helsinki; all subjects or their care-givers gave informed and written consent according to a protocol approved by the local ethics committee of the Don Carlo Gnocchi Foundation–ONLUS, Milan, Italy (#12_21/6/2018).

### SNPs typing

Whole blood was collected for all the subjects of the main study population and genomic DNA was isolated by phenol–chloroform extraction. Customer-design TaqMan^®^ probes for the 112 and 158 codons were used to determine the genotype of *APOE* [25].

*FCGRIIB* SNP rs1050501 (C > T) [26], was determined by a custom-designed TaqMan^®^ genotyping assay from Applied Biosystems Inc. (by Life Technologies, Foster City, CA, USA), using a two-step approach. First, using the following primers, a 494-base pair fragment was amplified:

Forward primer: 5′-CTAAGAGGAGCCCTTCCCTATGT-3′

Reverse primer: 5′-AATACGGGCCTAGATCTGAATGTG-3′

This was followed by a TaqMan RT-PCR using two probes specific for each allele (C and T). The primer and probe sequences for this reaction are listed below:

Forward primer: 5′-CCTAGCTCCCAGCTCTTCAC-3′

Reverse primer: 5′-CCACTACAGCAGCAACAATGG-3′

Reporter 1 (C-specific): HEX-TCACTGGGACTGCTGTAGCG-NFQ

Reporter 2 (T-specific): FAM-TCACTGGGATTGCTGTAGCG-NFQ

### Anti-HSV-1 IgG antibody measurements

For a subgroup of 170 subjects (69 AD, 52 MCI and 49 HC) serum samples were available for the detection of HSV-1 IgG titers, using a commercial enzyme-linked immunosorbent assay (ELISA) (IBL International, Hamburg, Germany). The optical densities (OD) were determined at 450 nm, using 620 nm reading as reference wavelength, as reported by datasheet. HSV-1 Ab titers were expressed as antibody index (AI), calculated by dividing OD measurement generated from the assay by OD cut-off calibrator. Quantitation of the four different HSV-1 IgG subclasses was carried out by a modified ELISA assay (IBL International), using four biotinylated subtype-specific monoclonal antibodies (Sigma-Aldrich, St. Louis, Mo, US), as previously described [27].

### Statistical analysis

Chi‐square goodness of fit test was used to verify that genotypes were in Hardy–Weinberg (HW) equilibrium and contingency. Chi square was used to evaluate differences between groups. *FCGRIIB* allelic polymorphism distribution in AD, MCI and HC was analyzed by odds ratio (OR) and 95% confidence interval (95% CI). p‐value was considered significant when < 0.05 after Bonferroni correction for two degrees of freedom (Pc) in 2 × 3 and 2 × 2 contingency tables.

The parametric data were expressed as mean ± standard deviation, whereas the non-parametric data as median and interquartile range (IQR). AD, MCI and HC were compared on demographic data using Chi square test and One-way ANOVA with Bonferroni post hoc test for categorical and continuous variables, respectively. Differences in experimental data among groups were tested using Kruskal–Wallis test and, when appropriate, the Mann–Whitney U test, and the correlations using Spearman’s correlation coefficient. The p-values corresponding to < 0.05 were described as statistically significant in the text. The statistical analyses were accomplished using commercial software (MedCalc Statistical Software version 14.10.2, Ostend, Belgium). A priori power analysis was run with the G-power software [28].

## Results

### Clinical characteristics

Demographic and clinical characteristics of the study population are summarized in Table 1. Gender and age were similar in all groups examined. As expected, the MMSE score was lower in AD (18.66 ± 5.53) compared to MCI (24.85 ± 2.89) (p < 0.01).

**Table 1:** Demographic, clinical characteristics and *FCGRIIB* rs1050501 genotypes distribution of subjects enrolled in the study

	AD	MCI	Healthy controls
N.	225	93	201
Gender (M:F)	81:144	49:44	80:121
Age, years	76.8 ± 6.4	75.0 ± 6.4	74.8 ± 10.3
MMSE	18.7 ± 5.5*	24.8 ± 2.9	–
APOE ε-4 carriers (%)	52.0^#,^°	39.5^#^	17.7
HSV-1 IgG (AI)^a^	8.9; 7.1–10.1**	8.8; 6.7–11.1***	7.4 6.0–8.5
HSV-1 Avidity index (%)^a^	87.9; 79.8–95.0	91.4; 85.7–97.6	90.1; 81.3–95.6
*FCGRIIB* rs1050501 genotypes (%)			
TT	78.7	75.3	77.1
CT	19.1	22.6	20.4
CC	2.2	2.1	2.5

Data are reported as mean ± standard deviation or as median; Interquartile range

*AD* Alzheimer’s disease, *MCI* Mild Cognitive Impairment, *M* male, *F* female, *MMSE* mini mental state evaluation, *APOE* Apolipoprotein E, *AI* antibody index

*p < 0.0001 compared to MCI

^#^p < 0.0001 compared to HC

°p = 0.046 compared to MCI

**p = 0.001 compared to HC

***p = 0.0077 compared to HC

^a^performed on a subgroup of 69 AD 52 MCI and 49 HC

### *APOE* and *FCGRIIB* genotype distribution

As expected, *ApoE4* variant was more frequently seen in AD (52.0%) and MCI (39.5%) patients compared to HC (17.7%) (p < 0.0001 for both), and in AD compared to MCI (p = 0.046). The genotype distribution of the *FCGRIIB* polymorphism was in HW equilibrium in the three groups of individuals enrolled in the study. Genotype and allelic distribution of *FCGRIIB* was comparable in the study population (Table 1). Moreover, we analyzed the *FCGRIIB* genotype distribution after categorization for *APOE4* (pos/neg) and sex (male/female) in the study population, but no differences were found (data not shown).

### Virological data

**Fig. 1:**
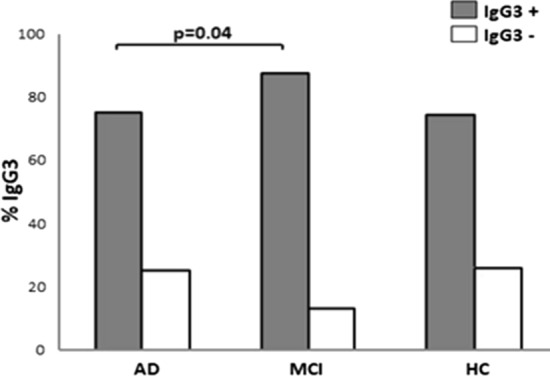
Frequency of HSV-1-specific IgG3 in 69 AD, 52 MCI and 49 HC subjects. The Chi square test was used to analyze the statistical differences among groups(AD vs. MCI: p = 0.04)

HSV-1 seropositivity was 97%, without difference among the three examined subgroups (95.6% for AD, 98.1% for MCI and 97.9% for HC). Results confirmed [29] that HSV-1 titers were significantly higher in AD (p = 0.001) and MCI patients (p = 0.008) compared to HC; HSV-1 avidity index was reduced, although not significantly, in AD compared to the other two groups of individuals (Table 1). The percentage of individuals showing serum HSV-1-specific IgG1, IgG2 and IgG4 was comparable in AD, MCI and HC individuals, whereas IgG3 were more frequently observed in MCI (87.2%) compared to AD (75.0%, p = 0.04) and HC (74.3%) (Fig. 1).

### *FCGRIIB* polymorphism

**Fig. 2:**
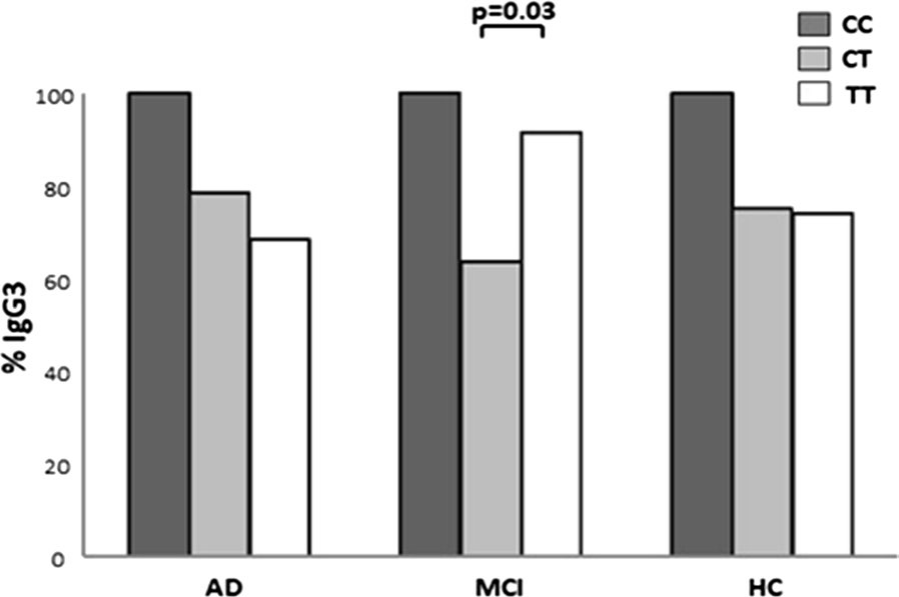
Frequency of HSV-1-specific IgG3 in 69 AD, 52 MCI and 49 HC subjects according to *FCGRIIB* rs1050501 genotypes distribution. The Chi square test was used to analyze the statistical differences among groups (MCI CT vs. MCI TT: p = 0.03)

No associations were detected between serum HSV-1 antibody and *FCGRIIB* genotypes when the entire study population or the three groups alone were analyzed (Table 2). No correlations were detected either between *FCGRIIB* genotypes and HSV-1-specific IgG1, IgG2 and IgG4 in the study population. In MCI though, the *FCGRIIB* TT genotype correlated with a significantly increased likelihood to observe HSV-1-specific serum IgG3 antibodies (94.1%), whereas MCI carrying CT genotype were less likely to present IgG3 antibodies (63.6%) (p = 0.03) (Fig. 2).

**Table 2:** *FCGRIIB* rs1050501 genotypes and corresponding anti-HSV1 IgG antibody index in AD patients, MCI subjects, and healthy controls

	*FCGRIIB* rs1050501 genotypes (%)	HSV-1 Ab IgG (AI)	HSV-1 IgG1 (OD)	HSV-1 IgG2 (OD)	HSV-1 IgG3 (OD)	HSV-1 IgG4 (OD)
AD (no 69)	TT (74.2)	8.94; 7.17–10.50	2.06; 1.47–2.56	0.95; 0.76–1.14	0.65; 0.49–0.78	0.59; 0.58–0.72
	CT (22.7)	8.78; 7.22–9.33	2.23; 1.13–2.44	0.75; 0.75–0.75	0.69; 0.49–0.86	0.58; 0.57–1.38
	CC (3.1)	7.60; 5.5–9.71	3.18; 3.18–3.18	–	0.46; 0.46–0.46	–
MCI (no 52)	TT (74.5)	8.82; 7.1–10.96	2.03; 1.68–2.60	0.66; 0.53-0.80	0.75; 0.47-0.95	0.58; 0.56–0.82
	CT (21.6)	8.03; 5.25–10.99	2.31; 2.02–3.07	–	0.61; 0.48–0.77	0.6; 0.57–1.04
	CC (3.9)	10.77; 8.85–12.7	2.29; 1.99–2.59	–	0.44; 0.42–0.47	–
HC (no 49)	TT (79.2)	7.53; 6.12–8.83	2.56; 1.98–2.99	–	0.62; 0.53–0.72	0.58; 0.54–1.81
	CT (18.7)	6.29; 5.60–7.74	2.46; 1.49–2.94	–	0.89; 0.36–1.17	1.02; 0.68–1.48
	CC (2.1)	7.47; 7.47–7.47	3.12; 3.12–3.12	–	–	0.55; 0.55–0.55

Data are reported as median; Interquartile range

*AD* Alzheimer’s disease, *MCI* Mild Cognitive Impairment, *AI* antibody index, *OD* optical density

## Discussion

We focused our attention on FCGRIIB, a receptor of the FCGR family that plays an important role in the immune cell activation, balancing immunoprotection and immunopathology [30]. FCGRs are glycoproteins that bind the Fc component of IgG. FCGRIIB length is 310 aminoacids, and because of its tertiary structure–with one intracellular, one extracellular and one transmembrane domain–the protein localizes in the plasma membrane [31, 32]. FCGRIIB is widely expressed on immune cells, including monocytes, neutrophils, macrophages, basophils, eosinophils and B-cells [33–35] and plays an inhibitory role, preventing an inappropriate activation of immune cells in the absence of antigens [15]. Interestingly, FCGRIIB is associated with the accumulation of amyloid beta in 3xTg-AD mice, suggesting its possible involvement in AD [22]. *FCGRIIB* is characterized by several polymorphisms and recent results indicated that the SNP rs1050501 (I232T), located in exon 5, is associated with autoimmune diseases [36]. Although no differences were observed in *FCGRIIB* genotype distribution in our study population, we found an association between rs1050501 and IgG3 distribution in MCI, *i.e.* MCI individuals carrying the *FCGRIIB* TT genotype were much more likely to express HSV-1-specific IgG3 antibodies compared those carrying the CT genotype.

HSV-1 evades the host immune response by binding all IgG subclasses, except for IgG3, with a viral Fc receptor. The more common detection of HSV-1-specific IgG3 in MCI suggests that these individuals try to suppress HSV-1 reactivation by increasing the expression of the IgG3 subclass, the only one which mounts an effective antiviral immune response. Results herein allow to speculate that in MCI the ability to block the viral reactivation is due not only to IgG3 production but probably depends on the *FCGRIIB* rs1050501 genotype.

Further analyses with a larger cohort, as well as a longer clinical follow-up to verify if and when MCI and in particular, MCI carrying *FCGRIIB* TT genotype, will develop AD, and if the correlation with IgG3 in these converted patients remains significant, will be necessary to further confirm these data.

Moreover, future studies are needed to shed light on the mechanism linking *FCGR* polymorphisms, in particular *FCGRIIB* rs1050501, and IgG3. Thus, it will be important to verify whether *FCGR* polymorphisms leading to aminoacidic variants (i.e. *FCGRIIB* rs1050501 to I232T variant) could affect the protein structural conformation, possibly influencing signalling pathways and Ab affinity for antigens.

## Conclusion

Findings of the present work offer further support for HSV-1 being a factor in the pathogenesis of AD: all together these results suggest, for the first time, the presence of an association between HSV-1 humoral immune responses and *FCGRIIB rs1050501* SNP in the setting of MCI and AD.

## Data Availability

The datasets generated and/or analysed during the current study are not publicly available due to privacy or ethical restrictions but are available from the corresponding author on reasonable request.

## Abbreviations

AD: Alzheimer’s disease
MCI: Mild cognitive impairment
HC: Healthy control
CNS: Central nervous system
HSV-1: Herpes simplex virus type 1
APOE: Apolipoprotein E
Ab: Antibodies
FCGRs: Gamma receptor for the Fc portion of IgG Abs
SNPs: Single nucleotide polymorphisms
OD: Optical densities
AI: Antibody index
HW: Hardy–Weinberg
OR: Odds ratio
IQR: Interquartile range
